# Quality of life related to residual snoring after adenotonsillectomy: a pilot study

**DOI:** 10.5935/1984-0063.20200093

**Published:** 2021

**Authors:** Rita Catia Brás Bariani, Thais Moura Guimaraes, Wilana Moura, Mario Cappellette Junior, Sergio Tufik, Gustavo A. Moreira, Reginaldo Raimundo Fujita

**Affiliations:** 1 Universidade Federal de São Paulo, Department of Otorhinolaryngology and Head and Neck Surgery - São Paulo - SP - Brazil.; 2 Universidade Federal de São Paulo, Departamento de Psicobiologia - São Paulo - SP - Brazil.; 3 Faculdade de Odontologia de Bauru, Departamento de Ortodontia - São Paulo - SP - Brazil.

**Keywords:** Sleep Apnea, Obstructive, Snoring, Indicators of Quality of Life, Tonsillectomy

## Abstract

**Introduction:**

Few studies have addressed long-term quality of life related to residual snoring after adenotonsillectomy. The aim of this study was to compare scores from the OSA-18 questionnaire between children with residual snoring and non-snoring children two or more years after adenotonsillectomy.

**Material and Methods:**

The sample comprised 25 children divided into two groups, a group of 14 snoring children, and a control group of 11 non-snoring children. The OSA-18 questionnaire was applied to the volunteers. In the control group, it was completed by the caregivers of the children, while in individuals with residual snoring it was completed by the caregivers of children in the presence of a doctor or dentist. A statistical comparison was made using a generalized linear model.

**Results:**

The snorer group had a higher total OSA-18 score, and a higher score in all five domains compared to the control group.

**Conclusion:**

Children with residual snoring two or more years after adenotonsillectomy may have a worse quality of life compared to the control group.

## INTRODUCTION

In childhood, sleep-disordered breathing is associated with a myriad of health problems that reinforce the need for early diagnosis and treatment^1^. The prevalence of obstructive sleep apnea (OSA) in children over 3 years of age varies from 7.2% to 34.2%^2^, whereas habitual snoring has a prevalence of 5-35%^3^.

The most common otorhinolaryngological condition in children with OSA is adenotonsillar hypertrophy, with or without allergic rhinitis, and mouth breathing^4^. The goldstandard treatment for OSA in children is adenotonsillectomy, which normalizes polysomnography and improves quality of life^5, 6, 7^. Some patients still require additional treatment using alternative methods such as corticosteroids, rapid maxillary expansion^8^, myofunctional therapy^9^, behavioral measures for weight loss and diet^10^, or CPAP^11^. The orthodontic treatment for correction of maxillomandibular abnormalities or mandibular retrusion has been shown to improve OSA^12^. In addition, the sequelae of chronic oral breathing need to be corrected by the global treatment, which should involve a multidisciplinary team of sleep medicine that include: otorhinolaryngologist, speech and/or orthodontic therapy to restore normal breathing and craniofacial growth patterns^13^.

Studies have demonstrated that adenotonsillectomy can have a positive effect on a number of different aspects of the condition, such as improving behavior, cognition, and quality of life of children with adenotonsillar hyperplasia^14, 15, 16, 17^. However, most studies assess any improvements immediately, or for only a few months, after surgery. Few studies have analyzed these results over the long-term, with periods equal to, or greater than one year. Lima Junior et al. (2008)^18^ reported that a positive impact on quality of life was maintained for about 15 months after surgery. Significant improvement was observed in sleep-related QOL and behavioral problems in children with OSA during long-term follow-up after adenotonsillectomy^19^.

Another interesting finding was that, while short-term and long-term benefits of surgery were achieved, the score for the domain of physical symptoms was higher in the long-term, implying that caregivers perceive some recurrence of physical symptoms after surgery in their children despite long-term improvement in quality of life^20^.

Based on these findings, clinicians should note the potential for long-term changes in certain domains of quality of life; therefore, children with OSA require both short-term and long- term quality of life follow up after surgery. OSA can persist after adenotonsillectomy in 21% to 75%^21, 22^. The possible factors that influence the failure of the surgery are patient comorbidities such as obesity, craniofacial anomalies, and nasal obstruction^21, 23^.

Patients with residual OSA continue to have a low quality of life^24^. However, few studies have addressed quality of life in patients with only residual snoring after late adenotonsillectomy surgery. SDB (respiratory sleep disorder) is widely studied due to its high prevalence, but it is not often considered in routine childcare visits^25, 26^.

Failure to treat children with OSA can lead to a range of impairments that include behavioral problems^27, 28^, increased cardiovascular dysfunction^29, 30^, and impaired growth and development^30^. One of the complications of OSA in children is the increased prevalence of neurodevelopmental^31, 32^. Children with primary snoring are also at risk of developmental disorders and behavioral disorders^32, 33^. Persistent snoring and nocturnal enuresis can affect interpersonal relationships and quality of life^34^. Therefore, the objective of this study is to evaluate the quality of life by the OSA-18 of children with residual snoring 2 years after adenotonsillectomy surgery and a control group without snoring complaints.

## MATERIAL AND METHODS

In this pilot study, the sample comprised 2 groups, one with snoring children at least 2 years after adenotonsillectomy surgery, and a control group without snoring. Four hundred caregivers of patients who had undergone adenotonsillectomy at Hospital São Paulo were interviewed. Children who continued to have snoring symptoms were recruited into the study. The inclusion criteria were children, boys and girls, aged between 5 and 12 years old. The exclusion criteria were children with cardiac, pulmonary, neuromuscular diseases, and chromosomal syndromes. This study was approved by the research ethics committee of the Universidade Federal de São Paulo, number 0698011806/2017. The clinical trial registration number is RBR- 463byn.

### Snoring group

The group comprised 14 snoring children who were selected from patients, attending the Outpatient Clinic of the Pediatric Otorhinolaryngology Department at the Escola Paulista de Medicina, Universidade Federal de São Paulo, who had previously undergone adenotonsillectomy (a minimum period of 2 years) and had a severe snoring complaint on most nights, or every night, over the previous four weeks (questionnaire OSA-18). All children in this group underwent an otorhinolaryngological clinical examination, nasofibroscopy, and polysomnography.

### Control group

The control group were selected from the Escola Paulistinha de Educação, an elementary school of education for Unifesp workers, and comprised 11 children selected at random who did not snore, according to the results of the OSA-18 questionnaire.

### Procedures

#### OSA-18 Questionnaire

The OSA-18 is a questionnaire (Supplementary Table) designed to be used with children, validated in Portuguese^34^, that assesses the quality of life in children with apnea^35^.

OSA-18 consists of 18 questions divided into 5 domains: sleep disorder, physical symptoms, emotional symptoms, daytime function and caregiver concerns, each item has a score of 7 points (1 - “never” to 7 - “always”)^34, 35, 36^. Values less than 60 have a mild impact on quality of life, 60 to 80 have a moderate impact on quality of life, and above 80 is considered a serious impact on quality of life.

In healthy volunteers, the caregivers completed the questionnaire, while in individuals with residual snoring it was carried out by the children’s caregivers, in the presence of an otorhinolaryngologist or orthodontist.

#### Polysomnography

Only patients with residual snoring underwent an overnight polysomnography exam. The polysomnography considered the following values: children with an obstructive apnea/hypopnea index (OAHI) >2/hour, or an obstructive apnea index (OAI) >1/hour, were considered abnormal, in addition to percutaneous oxyhemoglobin saturation (SpO_2_) <92%^37^ (Table 1).

**Table 1:** Snorer sample: polysomnography characteristics.

Variables	Snorer
Sleep efficiency	80.7 % ± 13.3
Sleep latency	45.1 % ± 40.4
Arousal index	7.8 % ± 3.7
AHI	1.4 (nº/h) ± 1.3
Obstructive AHI	1.1 (nº/h) ± 1.2
Central AHI	0.3 (nº/h) ± 0.4
RDI	1.5 (nº/h) ± 1.4
Baseline SpO_2_	96.6 % ± 1.1
Mean SpO_2_	95.7 % ± 1.4
Minimum SpO_2_	91% ± 3.3

Notes: AHI: Apnea-hypopnea index; RDI: Respiratory disturbance index; SpO2: Oxygen saturation.

#### Statistical analysis

Shapiro-Wilk tests were used to evaluate the distribution of the variables. Descriptive data were presented as means and standard deviations or absolute frequency, and data were analyzed using a generalized linear model (GzLM) test. For the categorical variables, a GzLM with binary logistic distribution was used, and gamma distribution was used for continuous variables. A *p*- value of =0.05 was considered significant.

## RESULTS

The sample comprised 25 children, 14 snorers after adenotonsillectomy, and 11 controls. The evaluation of the OSA-18 questionnaire was controlled by age values. There was no difference between groups in respect of the anthropometric absolute and z score, and sex (Table 2). However, the snorers were older than the control group. All domains of quality of life in the OSA-18 were worse in the snorer group than in the control group. As a result, the snorer group had the highest total OSA-18 score, as well as the highest score in all 5 domains compared to control. The overall average score for OSA-18 was 82.5 (severe impact), which was statistically significant compared to the control group (*p*>0.05). In the descriptive assessment of polysomnography, snorers had an average obstructive apnea/hypopnea index of 1.1±1.2 and a minimum saturation of 91% ±3.3 and a 95.7% ±1.4 (Table 3).

**Table 2:** Total sample characteristics.

Variables	Control	Snorers	*p*
Males, n(%)	11 (79)	14(56)	0.30
Age, mean(SD)	7.9 ± 1.2	9.7 ± 1.8	<0.01
BMI z-score, mean (SD)	2.0 ± 2.5	1.7 ± 2.8	0.71

Notes: GzLM test; SD: Standard deviation.

**Table 3:** Total sample: OSA-18 characteristics.

Variables	Control	Snorers	*p*
Sleep disturbance	5 ± 1.6	19.6 ± 5.6	<0.01
Physical symptoms	8.4 ± 6.8	18.2 ± 5.1	<0.01
Emotional symptoms	4.9 ± 2.8	13.4 ± 4.5	<0.01
Daytime function	6 ± 3.7	12.8 ± 6.0	<0.01
Caregiver concerns	6.3 ± 3.2	19.2 ± 7.9	<0.01
Total	30.5 ± 12.4	83.1 ± 20.7	<0.01

Note: GLZM test, adjusted for age.

## DISCUSSION

All domains of quality of life evaluated by the OSA-18 were worse in the snoring group after adenotonsillectomy compared to healthy individuals.

The highest mean scores of the OSA-18 were observed for the domains “sleep disturbance”, followed by “caregiver concerns”, and “physical symptoms”, which involve questions about the most common aspects of apnea, namely snoring, choking, restless sleep, mouth breathing, upper airway infection, difficult feeding, concern of the parents about the child’s health. Lee et al. (2015)^20^ in their study also scored for the domain of physical symptoms was higher in the long run, implying that caregivers perceive some recurrence of physical symptoms after surgery in their children despite the long-term improvement in quality of life. However, Mitchell et al. (2004)^38^ found results similar to our study also in the domains sleep disorders and physical suffering were significantly lower (*p*. 005) in the short-term than in the long-term.

This study is relevant, because the impact of residual snoring on the quality of life of children who had undergone adenotonsillectomy 2 or more years previously is of great importance. It was observed that in all domains of quality of life evaluated by the OSA-18 were worse in the snoring group after adenotonsillectomy compared to healthy individuals. These results should alert all professionals who deal with patients with similar profiles to check for the presence of snoring in the long-term postoperative period and to seek treatment alternatives.

The data found in this study can contribute to the reevaluation and retreatment of these children to improve their quality of life. It is interesting to note that there are many studies that show that children with primary snoring present changes in their quality of life and more behavioral problems when compared to the control groups^39, 40, 41, 42, 43^. However, our study uses a sample of children at least 2 years after the adenotonsillectomy who have signs and symptoms of OSA. Also a systematic review demonstrated the effectiveness of adenotonsillectomy in improving the quality of life of pediatric patients with OSA^44^. All studies involving short-term follow-up (=6 months) have shown improvements in quality of life scores after adenotonsillectomy compared to preoperative values and studies involving long-term follow-up (>6 months) have shown mixed results. We must consider the low educational level and socioeconomic level of parents or guardians and their difficulty in accessing specialized services of the Brazilian health system (SUS). Children who continued to have snoring symptoms were recruited into the study.

Residual snoring should be thoroughly investigated in clinical practice, especially after adenotonsillectomy, as quality of life may be compromised in these children.

Quality of life has been increasingly seen as an important health outcome. OSA-18 is an easy and fast test, with high reliability and consistency, to assess the subjective aspects of quality of life^7^. This questionnaire, when combined with other clinical and objective parameters, allows professionals to better assess the impact of OSA on children and their family life, and to select the best type of treatment^24^. Being a subjective tool, it should be used as an adjunct to clinical examination to improve the diagnosis of pediatric OSA and not a replacement for pediatric polysomnography^7^.

The presence of nasal disuse and breathing through the mouth are abnormal functions that cause developmental and functional changes that are associated with the development of DRS. The impact of mouth breathing promotes changes in craniofacial growth patterns and how these changes lead to impaired developmental functions and the consequent persistence of DRS. Understanding the dynamics that lead to the development of DRS and recognizing the factors that affect craniofacial growth and the resulting functional impairments, allows for an adequate multidisciplinary treatment planning for sleep apnea in childhood. The increase in lymphoid tissue may actually be a consequence and not a cause of these initial dysfunctions^45^.

This study is unique for investigating long-term (>2 years) quality life of children submitted to adenotonsillectomy and the comparison with a control group. However, is has some limitations, including small sample size, limited spectrum of the condition, and a non-surgical control group.

As the effect size of the difference between groups were huge, the small sample size did not affect the final results.

Were choose to have a healthy control group, instead of a post-surgical, in order to have a purer comparison. Yet, a study comparing children post-adenotonsillectomy with and without residual snoring still need to be done. Confirmatory results are essential, and the repetition of studies by other researchers, replicating the conditions described, will produce the evidence for the advancement of science.

## CONCLUSION

The data suggest that snoring patients two or more years after adenotonsillectomy surgery have worse quality of life compared to the control group in long-term follow-up.

## OSA-18 for children with OSAS - English version.

**OSAS Quality of Life Survey (OSA-18) Date: _ /_ /_**

**Name: ___**

**For each question below, please circle the number that best describes how often each symptom or problem has occurred during the past 4 weeks. Please circle only one number per question. Thank you.**

**Table:**

	None of the time	Rarely any of the time	A little of the time	Some of the time	A good bit of the time	Most of the time	All of the time
**Sleep Disturbance**							
**During the past 4 weeks, how often has your child had...**							
loud snoring?	1	2	3	4	5	6	7
breath holding spells or pauses in breathing at night?	1	2	3	4	5	6	7
choking or made gasping sound while asleep?	1	2	3	4	5	6	7
restless sleep or frequent awakenings from sleep?	1	2	3	4	5	6	7
**Physical Symptoms**							
**During the past 4 weeks, how often has your child had...**							
mouth breathing because of nasal obstruction?	1	2	3	4	5	6	7
frequent colds or upper respiratory infections?	1	2	3	4	5	6	7
nasal discharge or a runny nose?	1	2	3	4	5	6	7
difficulty in swallowing foods?	1	2	3	4	5	6	7
**Emotional Symptoms**							
**During the past 4 weeks, how often has your child had...**							
mood swings or temper tantrums?	1	2	3	4	5	6	7
aggressive or hyperactive behavior?	1	2	3	4	5	6	7
discipline problems?	1	2	3	4	5	6	7
**Daytime Function**							
**During the past 4 weeks, how often has your child had...**							
excessive daytime sleepiness?	1	2	3	4	5	6	7
aggressive or hyperactive behavior?	1	2	3	4	5	6	7
discipline problems?	1	2	3	4	5	6	7
**Caregiver Concerns**							
**During the past 4 weeks, how often have the problems described above.**							
caused you to worry about your child's general health?	1	2	3	4	5	6	7
created concern that your child is not getting enough air?	1	2	3	4	5	6	7
interfered with your ability to perform daily activities?	1	2	3	4	5	6	7
made you frustrated?	1	2	3	4	5	6	7

## Portuguese version of OSA-18 (OSA-18-pv)

**OSA-18 Versão Portuguesa (OSA-18-pv) Data: _ / _ / _**

**Nome: ___**

**Em cada uma das questões seguintes, faça por favor um círculo à volta do número que melhor descreve a frequênci de cada sintoma ou problema nas últimas 4 semanas. Assinala apenas um número por questão. Obrigado.**

**Table:**

	Nunca	Quase Nunca	Poucas Vezes	Algumas Vezes	Bastantes vezes	Quase Sempre	Sempre
Distúrbio do Sono							
Nas últimas 4 semanas, com que frequência o seu filho teve...							
ressonar alto?	1	2	3	4	5	6	7
paragens na respiração durante a noite?	1	2	3	4	5	6	7
engasgos ou respiração ofegante enquanto dormia?	1	2	3	4	5	6	7
sono agitado ou despertares frequentes do sono?	1	2	3	4	5	6	7
**Sintomas Físicos**							
**Nas últimas 4 semanas, com que frequência o seu filho teve...**							
respiração bucal por obstrução nasal?	1	2	3	4	5	6	7
resfriados ou infecções das vias aéreas superiores?	1	2	3	4	5	6	7
secreção e congestão nasal?	1	2	3	4	5	6	7
dificuldade em engolir alimentos?	1	2	3	4	5	6	7
**Problemas emocionais**							
**Nas últimas 4 semanas, com que frequência o seu filho teve...**							
alterações do humor ou acessos de raiva?	1	2	3	4	5	6	7
comportamento agressivo ou hiperactivo?	1	2	3	4	5	6	7
problemas disciplinares?	1	2	3	4	5	6	7
Problemas do quotidiano							
Nas últimas 4 semanas, com que frequência o seu filho teve...							
sonolência diurna excessiva?	1	2	3	4	5	6	7
episódios de falta de atenção ou concentração?	1	2	3	4	5	6	7
dificuldade ao levantar da cama de manhã?	1	2	3	4	5	6	7
**Opinião do Informante**							
**Nas últimas 4 semanas, com que frequência os problemas acima descritos...**							
causaram preocupação com a sua saúde?	1	2	3	4	5	6	7
preocuparam-no pelo seu filho não poder respirar ar suficiente?	1	2	3	4	5	6	7
interferiram com as suas actividades diárias?	1	2	3	4	5	6	7
deixaram-no frustrado?	1	2	3	4	5	6	7

## References

[1] Schechter MS (2002). Technical report: diagnosis and management of childhood
obstructive sleep apnea syndrome. Pediatrics.

[2] Izu SC, Itamoto CH, Pradella-Hallinan M, Pizarro GU, Tufik S, Pignatari S (2010). Obstructive sleep apnea syndrome (OSAS) in mouth breathing
children. Braz J Otorhinolaryngol.

[3] Petry C, Pereira MU, Pitrez PM, Jones MH, Stein RT (2008). The prevalence of symptoms of sleep- disordered breathing in
Brazilian schoolchildren. J Pediatr (Rio J).

[4] Chervin RD, Ruzicka DL, Hoban TF, Fetterolf JL, Garetz SL, Guire KE (2012). Esophageal pressures, polysomnography, and neurobehavioral
outcomes of adenotonsillectomy in children. Chest.

[5] Guilleminault C, Huang YS, Glamann C, Li K, Chan A (2007). Adenotonsillectomy and obstructive sleep apnea in children: a
prospective survey. Otolaryngol Head Neck Surg.

[6] Tauman R, Gulliver TE, Krishna J, Montgomery-Downs HE, O’Brien LM, Ivanenko A (2006). Persistence of obstructive sleep apnea syndrome in children after
adenotonsillectomy. J Pediatr.

[7] Sistla SK, Lahane V (2019). OSA 18 questionnaire: tool to evaluate quality of life and
efficacy of treatment modalities in pediatric sleep disordered breathing due
to adenotonsillar hypertrophy. Indian J Otolaryngol Head Neck Surg..

[8] Lagravere MO, Major P W, Flores-Mir C (2005). Long-term dental arch changes after rapid maxillary expansion
treatment: a systematic review. Angle Orthod.

[9] Wu LM, Wu XF, Yu ZM, Liu Y (2017). Systematic review on orofacial myofunctional therapy to treat
obstructive sleep apnea-hypopnea syndrome. Lin Chung Er Bi Yan Hou Tou Jing Wai Ke Za Zhi.

[10] Friedman M, Wilson M, Lin HC, Chang H W (2009). Updated systematic review of tonsillectomy and adenoidectomy for
treatment of pediatric obstructive sleep apnea/hypopnea
syndrome. Otolaryngol Head Neck Surg.

[11] Marcus CL, Brooks LJ, Draper KA, Gozal D, Halbower AC, Jones J (2012). Diagnosis and management of childhood obstructive sleep apnea
syndrome. Pediatrics.

[12] Huynh NT, Desplats E, Almeida FR (2016). Orthodontics treatments for managing obstructive sleep apnea
syndrome in children: a systematic review and meta-analysis. Sleep Med Rev..

[13] Nishikawa H, Pearman K, Dover S (2003). Multidisciplinary management of children with craniofacial
syndromes with particular reference to the airway. Int J Pediatr Otorhinolaryngol.

[14] Serres LM, Derkay C, Astley S, Deyo RA, Rosenfeld RM, Gates GA (2000). Measuring quality of life in children with obstructive sleep
disorders. Arch Otolaryngol Head Neck Surg.

[15] Serres LM, Derkay C, Sie K, Biavati M, Jones J, Tunkel D (2002). Impact of adenotonsillectomy on quality of life in children with
obstructive sleep disorders. Arch Otolaryngol Head Neck Surg.

[16] Nascimento GMS, Salgado DC, Maia MS, Lambert EE, Pio MRB, Tiago RSL Impacto do tratamento cirúrgico na qualidade de vida de
crianças com hiperplasia de tonsilas. Acta ORL.

[17] Van Staaji BK, Van Den Akker EH, Rovers MM, Hordijk GJ, Hoes AW, Schilder AG (2005). Effectiveness of adenotonsillectomy in children with mild
symptoms of throat infections or adenotonsillar hypertrophy: open,
randomised controlled trial. Clin Otolaryngol.

[18] Lima Júnior JM, Silva VC, Freitas MR (2008). Long term results in the life quality of children with
obstructive sleep disorders submitted to adenoidectomy/
adenotonsillectomy. Braz J Otorhinolaryngol.

[19] Song IS, Hong SN, Joo J W, Han MS, Hwang SJ, Seo MY (2020). Long-term results of sleep-related quality-of-life and behavioral
problems after adenotonsillectomy. Laryngoscope.

[20] Lee CH, Kang KT, Weng WC, Lee PL, Hsu WC Quality of life after adenotonsillectomy in children with
obstructive sleep apnea: short-term and long-term results. Int J Pediatr.

[21] (2015). Otorhinolaryngol.

[22] Imanguli M, Ulualp SO (2016). Risk factors for residual obstructive sleep apnea after
adenotonsillectomy in children. Laryngoscope.

[23] Pomerantz J. Management of persistent obstructive sleep apnea after
adenotonsillectomy. Pediatr Ann.

[24] Tauman R, Gulliver TE, Krishna J, Montgomery-Downs HE, O’Brien LM, Ivanenko A (2006). Persistence of obstructive sleep apnea syndrome in children after
adenotonsillectomy.

[25] Silva VC, Leite AJM (2006). Quality of life in children with sleep-disordered breathing:
evaluation by OSA-18. Braz J Otorhinolaryngol.

[26] Ali N, Pitson D, Stradling J (1993). Snoring, sleep disturbance, and behaviour in 4-5 year
olds. Arch Dis Child.

[27] Nieminen P, Tolonen U, Löppönen H (2000). Snoring and obstructive sleep apnea in children: a 6month
follow-up study. Arch Otoloryngol Head Neck Surg.

[28] Bruni O (2010). The importance of sleep for children’s well being. Sleep Med.

[29] O’Brien LM, Mervis CB, Holbrook CR, Bruner JL, Klaus CJ, Rutherford J (2004). Neurobehavioral implications of habitual snoring in
children. Pediatrics.

[30] American Academy of Sleep Medicine (AASM) (2014). International classification of sleep disorders.

[31] Gozal D, Pope DW (2001). Snoring during early childhood and academic performance at ages
thirteen to fourteen years. Pediatrics.

[32] O’Brien LM, Holbrook CR, Mervis CB, Klaus CJ, Bruner JL, Raffield TJ (2003). Sleep and neurobehavioral characteristics of 5- to 7-year-old
children with parentally reported symptoms of attention-deficit/
hyperactivity disorder. Pediatrics.

[33] Tsara V, Amfilochiou A, Papagrigorakis JM, Georgopoulos D, Liolios E, Kadiths A (2010). Guidelines for diagnosing and treating sleep related breathing
disorders in adults and children (Part 3: obstructive sleep apnea in
children, diagnosis and treatment). Hippokratia.

[34] Uema SFH, Pignatari SSN, Fujita RR, Moreira GA, Pradella-Hallinan M, Weckx L (2007). Assessment of cognitive learning function in children with
obstructive sleep breathing disorders. Braz J Otorhinolaryngol.

[35] Fernandes FMVS, Teles RCVV (2013). Questionário da síndrome da apneia obstrutiva na
criança- 18: versão portuguesa. Braz J Otorhinolaryngol.

[36] Franco RA, Rosenfeld RM, Rao M (2000). First place--resident clinical science award 1999. Quality of
life for children with obstructive sleep apnea. Otolaryngol Head Neck Surg.

[37] Baldassari CM, Mitchell RB, Schubert C, Rudnick EF (2008). Pediatric obstructive sleep apnea and quality of life: a
meta-analysis. Otolaryngol Head Neck Surg.

[38] Marcus CL, Moore RH, Rosen CL, Giordani B, Garetz SL, Taylor G (2013). A randomized trial of adenotonsillectomy for childhood sleep
apnea. N Engl J Med.

[39] Mitchell RB, Kelly J, Call E, Yao N (2004). Long-term changes in quality of life after surgery for pediatric
obstructive sleep apnea. Arch Otolaryngol Head Neck Surg.

[40] Crabtree VM, Varni J W, Gozal D (2004). Health-related quality of life and depressive symptoms in
children with suspected sleep-disordered breathing. Sleep.

[41] Goldstein NA, Fatima M, Campbell TF, Rosenfeld RM (2002). Child behavior and quality of life before and after tonsillectomy
and adenoidectomy. Arch Otolaryngol Head Neck Surg.

[42] Montgomery-Downs HE, Crabtree VM, Gozal D (2005). Cognition, sleep and respiration in at-risk children treated for
obstructive sleep apnoea. Eur Respir J.

[43] Blunden S, Lushington K, Kennedy D, Martin J, Dawson D (2000). Behavior and neurocognitive performance in children aged 5-10
years who snore compared to controls. J Clin Exp Neuropsychol.

[44] Honaker SM, Street A, Daftary AS, Downs SM (2019). The use of computer decision support for pediatric obstructive
sleep apnea detection in primary care. J Clin Sleep Med.

[45] Todd CA, Bareiss AK, McCoul ED, Rodriguez KH (2017). Adenotonsillectomy for obstructive sleep apnea and quality of
life: systematic review and meta-analysis. Otolaryngol Head Neck Surg.

[46] Guilleminault C, Akhtar F (2015). Pediatric sleep-disordered breathing: new evidence on its
development. Sleep Med Rev.

